# Proposal for an IIoT Device Solution According to Industry 4.0 Concept

**DOI:** 10.3390/s22010325

**Published:** 2022-01-02

**Authors:** Andrea Vaclavova, Peter Strelec, Tibor Horak, Michal Kebisek, Pavol Tanuska, Ladislav Huraj

**Affiliations:** 1 Institute of Applied Informatics, Automation and Mechatronics, Faculty of Materials Science and Technology in Trnava, Slovak University of Technology in Bratislava, 91724 Trnava, Slovakia; andrea.vaclavova@stuba.sk (A.V.); peter.strelec@stuba.sk (P.S.); michal.kebisek@stuba.sk (M.K.); pavol.tanuska@stuba.sk (P.T.); 2 Department of Applied Informatics, University of SS. Cyril and Methodius, 91701 Trnava, Slovakia; ladislav.huraj@ucm.sk

**Keywords:** Industrial IoT, OPC UA, IIoT design, Industry 4.0

## Abstract

Today, Industrial Internet of Things (IIoT) devices are very often used to collect manufacturing process data. The integration of industrial data is increasingly being promoted by the Open Platform Communications United Architecture (OPC UA). However, available IIoT devices are limited by the features they provide; therefore, we decided to design an IIoT device taking advantage of the benefits arising from OPC UA. The design procedure was based on the creation of sequences of steps resulting in a workflow that was transformed into a finite state machine (FSM) model. The FSM model was transformed into an OPC UA object, which was implemented in the proposed IIoT. The OPC UA object makes it possible to monitor events and provide important information based on a client’s criteria. The result was the design and implementation of an IIoT device that provides improved monitoring and data acquisition, enabling improved control of the manufacturing process.

## 1. Introduction

**Figure 1:**
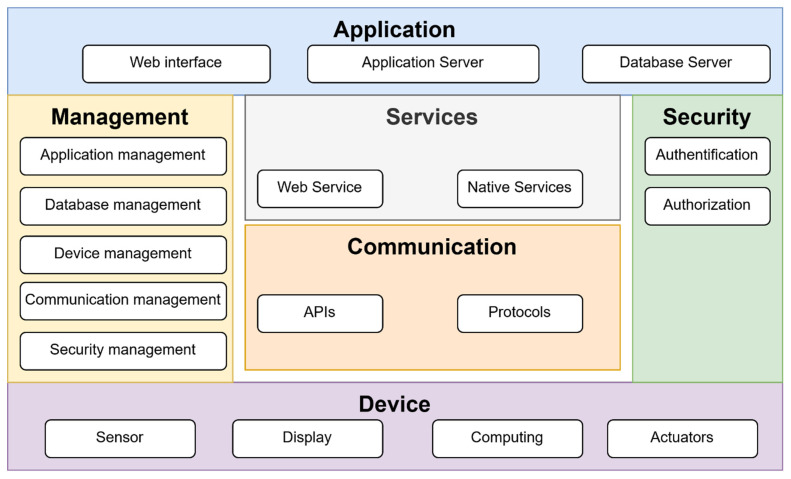
Model of IoT device: functional view specification.

The proposed IoT device is intended for home use. This type of design is based on the functional view specification shown in Figure 1. In this proposal, the layers responsible for the different parts of the application are introduced.

The application layer includes servers that implement the core services. Services provides the features that the IoT device supports. The communication layer defines the APIs and used protocols. The management layer is used to deal with application setup and operation. Security defines authentication and authorization. The device sections contain descriptions of sensors, displays and actuators, and computing. This approach defines the basic parts and their interconnection in IoT devices [1].

**Figure 2:**
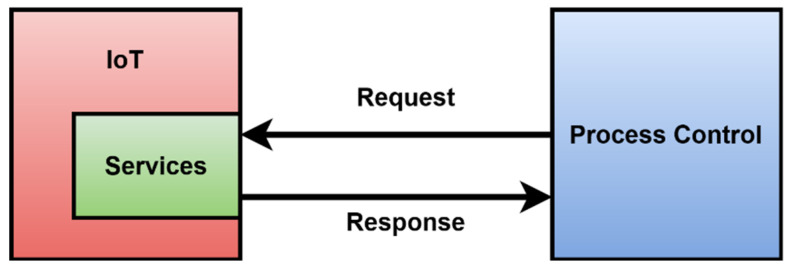
Model of common integration IoT in industry.

IoT design does not allow unambiguous identification of events, the description of states, and machine readable representation of the functionality. IoT device design is a tradeoff between performance and functionality. Its integration may not be straightforward. The default integration model of an IoT device is shown in Figure 2.

The design of IoT devices is based on a proposal, which is oriented to provide standard software development. Figure 2 shows a typical case of integration. Process control triggers data periodically, and data are acquired from the IoT. Commonly, a request response protocol is used. For IoT that use the published subscribe protocol, it is possible that an IoT device can send data and publish to consumers. During the development of Industry IoT devices, there were limits of design detected. The standard proposal for IoT devices is described in the following paragraphs.

The purpose and requirement of hardware and software introduce a set of functionally oriented requirements, such as data collection, data analysis requirements, system administration, and what level of personal data protection should be there; it is appropriate to evaluate the sensitivity of data and related security requirements. Moreover, it includes user interface requirements [2].

The process specification contains the requirements of each phase of the function. In this section, it is appropriate to create cases using unified modeling language (UML) of the IoT system and to formally describe the purpose and any specified requirements for the device [3].

A domain model is a representation of objects regardless of their data structures, and it represents all objects in the system.

An information model represents concepts and relationships, constraints, rules, and operations to determine the semantics of data for the selected domain [4,5]. In this part, it is unimportant how the information is represented or stored, but it appends attributes and relationships, or it defines newly created virtual entities.

A service specification is the level where processes and information models are mapped to individual services.

An IoT-Level specification specifies a level at which the IIoT device will operate. All these levels need an independent analysis for their implementation, where it is possible to clearly represent the functionalities of the device [6].

The integration is described in Figure 2. An IoT integration is based on data generated on the production line model [7].

The application and hardware (HW) development are chosen, based on the prior technical specification.

The noted proposal was commonly used for developing IoT devices utilising services. A service provided data acquired from the sensors. IoT device integration is limited due to the small amount of supported protocols and limited computing power. Usually, there is a need to run applications such as a broker, proxy, or application layer provided by the manufacturing execution system (MES) to support the connectivity of the IoT device [8]. The IoT itself does not provide a model of internal functionality in the noted design. Data acquired from IoT are missing additional parameters such as state, timestamp, or quality assessment. The acquired data can provide an advantage for production, but there is no generated event that can be integrated into process control [9].

IoT security and data quality are also important [10]. Solutions include blockchain technology for recording the attribute, avoiding data tampering, and eliminating a single point of failure at edge computing devices [11]. The concepts of security, authentication, and authorization are missing as well.

### 1.1. Motivation for This Article

In industry, IoT device integration is possibly based on the supported protocols. If the protocol is not supported, it is necessary to provide data transformation [12]. One option is to deploy an OPC UA server and client that can map the desired values to the IoT device [13]. After experience with IoT device design and deployment, shortcomings have been identified. In the absence of event generation, measured values did not have a defined time of occurrence, and their validity and limit of values, inability to retrieve historical data, and set control parameters were all limiting factors. After analysis, it was identified that there is a need to provide these parameters for Industrial IoT. The current article modifies the proposal to approach the design of IIoT devices for industry. The framework should support the design of an Industry IoT that can be easily applied and create an information model according to the OPC UA standard. The proposal should approximate the inner workings of the device and ensure the generation of events usable for manufacturing process control and monitoring.

Manufacturing companies are investing significantly in the Industry 4.0 data and infrastructure of their production lines. However, many of these systems still leave a gap between the collection of production data and actions that can be taken with them [14]. Big data and data mining on production data can identify any deficiencies in the production process [15]. The data, produced by the production lines, are represented as a record, or a whole set of records, and are used to store automatically produced documentation of events, behavior, and activities of the system [16].

Data flow is the transmission of data between devices. A large number of devices produce a data stream that is composed of smaller data chunks [17]. Traditionally, batch processing has been the primary method of data processing where large volumes of data are acquired at fixed intervals [18]. This affects the timeliness of data.

Analysis and evaluation of data produced by a production line shows the issues in the control process [19]. Then, it is possible to consider improving the efficiency or quality there [20]. The addition of new measured values or the possibility of better regulation, by using IIoT devices, enables minimizing the risk of changing the production process [21].

When integrating or tracking existing data, the resulting data streams work well if the goal is to identify data patterns for time events, such as device telemetry, or geolocation, generated events, and transmission monitoring [22]. Streaming analytics is the processing and analysis of data records continuously [23]. With event-driven architectures and integration of IIoT devices, there is the basic possibility to improve control of processes or obtain information, which is important for predictive maintanance [24]. Event streaming is the practice of capturing data in realtime [25]. Event streaming thus ensures a continuous flow and interpretation of data. IoT devices do not provide event streaming data. They are characterized by the fact that they generate large volumes of heterogeneous sensor data [26]. There are many variants of IoT devices within the communication protocols used by the connectable sensors. [27]. IoT devices cannot provide historical data of the measured values. Integration of IoT into production lines controlled by an MES system can acquire data from an IoT device. This process can acquire data from all elements of a production line and save data into a relational database or process that is allocated for data analyses [28].

There were identified several issues related to IoT and the data acquisition process. Data that are not correctly acquired are represented by a record that indicates that this activity is finished with an error status or poor quality. IIoT devices can be obstacles, and, therefore, it is good to evaluate the impact on control or measurement [29]. In practice, there is a possibility to simulate data delivery for repetitive read activities. Such a scenario is used in noncritical parts of the production line. Temperature may be unsuccessfully read, but the last value from a few second ago is good enough. Such cases are handled by previous successful reads, and the process appears to have taken place without direct impact on the production line. These features could be included in the design of the IIoT device, if necessary, if the solution used as a digital twin is taken in to consideration [30,31,32].

These shortcomings are negligible for the description of the production process. However, for the needs of analysis or creation of a simulation model of the production process, these data are insufficient. The data obtained from the MES system are also not in a good state for further use in terms of simulations [33,34].

A treatment and more detailed examination in the event of such a failure to write data to the database are both necessary to perform an additional analysis of other sources and data, of course, if it is possible [35]. These are called log files, where it is possible to identify insufficient data and other properties that affect the data recording process, such as system usage, system resource, occupancy, and others [36].

By integrating these data into the data from the production line, it is possible to identify the reasons and the real source of error events [37]:

1. Prevention of error events: here, an identification of the specific states when errors occur is required.
2. Ensuring the flow of the process: in cases when the flow is not well known, process mining is required [38,39].

**Figure 3:**
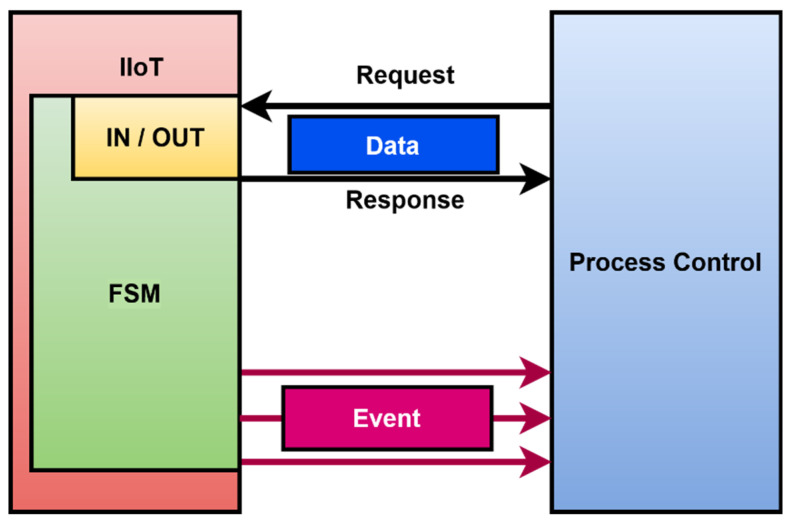
Model industry IoT device and its integration.

The result of the analysis of the acquired data is the identification of issues. The understanding of the control process will allow the definition of the goal that the IIoT device should reach. All these facts lead into the model shown in Figure 3. This model is oriented to provide more data, and it is able to generate or trigger events.

This is a major change in the understanding of an IoT device and its function in the production line. One advantage is internal usage of a finite state machine. The advantage is that in industry this is well known, and there are technologies where FSM is used for the representation of functionality. This standard is OPC UA.

OPC UA is a comunication protocol. It is also set of standards that are used for integration in industry. It is ready to support 5G networks, which provide credible schemes, such as the high quality of service, ultra-low latency, and improved security over the pre-existing architectures [40].

Such a model fit into the OPC UA objects can be used for better design of Industry IoT devices. Comparision of the IoT models shown in Figure 1 and Figure 3 shows the basic differences. The standard IoT is service-oriented. There is no internal representation of the device. It is impossible to send any aditional information other than the data, which are already prepared. There is no guaranted time of read data. An industry IoT device based on the model shown in Figure 3 is able to provide data in the standard way as an IoT device. There are exactly allocated inputs and outputs. OPC UA is an industry protocol that provides precision data with attributes such as a timestamp and the quality of the data, which the standard IoT is not able to cover [41]. Events are generated during the execution of each state and can be provided for process control. Events can be used for controlling the process, internal debugging, and signaling and monitoring. All these facts are more suitable to obtain precision data from a production line and discover new knowledge in control. Such design of an IIoT device supports big data, and the acquired data can be used for adaptation and increasing quality in process control [42].

### 1.2. Problem Statement and Contribution

Given the widespread use of IIoT devices in industrial areas, the issues of data collection and integration also need to be addressed. One of the frequently used methods is based on the use of industry standard OPC UA. These standards emphasize compatibility and an open platform within the Industry 4.0 concept. Existing IIoT devices are incompatible with these industry standards. This paper presents a description of the development of an IIoT device that offers the compatibility and benefits of OPC UA. The created device can use attributes of OPC UA such as events, data read and write, and internal representation of the device as a model represented by FSM, etc.

The main advantages of the proposed device include the use of events. Unlike conventional IIoT devices, they enable improved control of manufacturing processes by responding to events that occur during these manufacturing processes. Another advantage of the proposed IIoT device is the possibility of dynamically changing its configuration and data transmission and expanding the parameters of the collected data. The data can be acquired with attributes of quality, accuracy, time stamps, etc. Using data and events is already possible during the development of the IIoT device itself. Testing and debugging of the device is facilitated by being able to check or change states and read and write test data. This allows better debugging of the device and thus speeds up development and identification of potential issues.

The proposed IIoT device enables exporting and visualizing the internal functionality in an FSM model. This allows a better understanding of the device behavior and, thus, facilitates its integration into the manufacturing process. This capability is completely missing in the standard IIoT. The proposed IIoT device is compatible with control systems of different manufacturers. This will ensure easier integration and use in multiple hierarchical control layers.

The result is the design and implementation of an IIoT device that provides improved monitoring and data acquisition, enabling improved control of the manufacturing process.

### 1.3. State of the Art

OPC UA is suitable for integration of almost any component, but several hardware components do not support it by default in IoT [43]. There are few devices supporting this standard. Many IoT devices used message queue telemetry transport (MQTT) protocol by default but without encyption and basic security possibilities. OPC UA is able to provide the same functionality and make a device compatible and easy for integration in a production line [44].

The OPC UA base node provides a basic description of what an IIoT device should cover and which parts are important in the design of an IIoT.

The basic structure in the OPC UA is the node. An address space has all nodes, their attributes, and properties. That means all nodes are allocated in the address space. Everything in the OPC UA address space is represented as a node.

**Figure 4:**
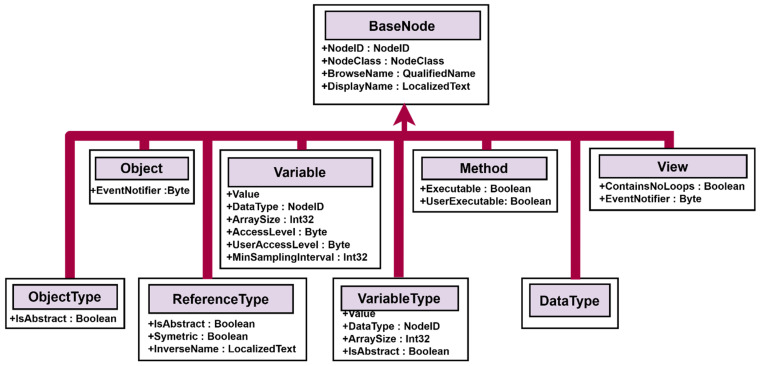
OPC unified architecture basenode structure.

Nodes are connected via references. A reference is again just a node that describes how nodes are connected with each other [45]. OPC UA defines eight basic nodes, which are nonextensible, and each of them has a defined set of attributes, as is shown in Figure 4.

The design of the information model standard is based on define types. Define types can be object and variables type, reference type, and data types. There are existing standard methods, and they must contain basics, such as start and stop functionality, and this must be implemented by default.

Next, the parameters are definitions of properties, modeling rules, and encoding for data types if they are needed. More information can be found in the specification [46].

It is important to distinguish between nodes and objects. Nodes originate from different node classes, and they are described by attributes defined in node classes. Nodes can have properties from other nodes. Nodes are connected to each other via references. References are derived from the reference types and they are mapping connections between different nodes [47]. Objects are nodes in a certain node class. Objects can include variables, methods, events, or other objects. The object is represented, including the components, in the address space. The next component is a variable. A variable represents a value. There can be limits for a value, and they are also defined. For nonstandard variables, they can define their own. Nevertheless, all variables are defined by variable type [48].

Variable type specifies variables and the definition of a template for instancing variables. It also specifies a group of components that are valid for each variable type. A data type is an attribute, a variable, and variable type. One of them is mandatory. The data type can be scalar or it can be represented by a complex structure [49].

Based on these facts, it was identified that the standard design of an IoT is oriented to achieve a working system, and there is no need to consider internal processes, historical data, or creation of an information model of the device. Therefore, the design of IoT with easy functionality did not fit with a better structured design of the device, which was close to unified architecture (UA) specification [50].

**Figure 5:**
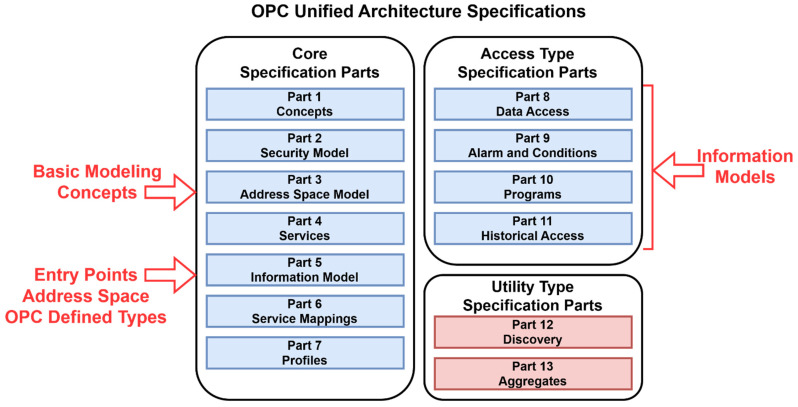
OPC unified architecture specifications.

OPC UA specification is shown in Figure 5. The important parts for the design of an IIoT are spread via more parts. Core specification is an important basic modeling concept and information model. The primary objective of the OPC UA address space is to provide a standard way for OPC UA servers to represent objects to OPC UA clients.

The information model represents the functionality of the device. This fact leads into the idea to design the IoT as a finite state machine. A finite state machine is closer to the functionality of an industry device. It is also possible to use a standard proposal, but then there should be integration with a basic IoT via some external OPC UA server. Such a solution is unable to cover all the advantages that OPC UA can provide. Nevertheless, it is possible to integrate an IoT via an external OPC UA server and use it in a production line [51,52].

However, this has limited usage, and it can only provide data access and connectivity with IoT devices. This limitation was the next reason to obtain more detail for the design. There are three main goals that need to be achieved. The first one is data access and, in particular, historical data access. The second one is to have available events and the possibility of using them in the control systems. The third one is basic security with authentification and authorization, which is always the weak part of standard IoT devices.

Our research started to transform use cases into a useful specification and convert it into an FSM. Business Process Model and Notation (BPMN) provides a graphical syntax to capture the use cases but does not cover the state of the modeled entities [53,54].

## 2. Materials and Methods

MES systems represent a set of tools that refine the management of the production process, and, with their extended possibilities, improve the quality of production and enable correct decision making [55]. An MES system is also an integrated system, which enables the flow of information in the company, or it directly provides interventions and the possibility of managing lower levels.

This was stimuli for proposal of the IIoT solution. The proposal was implemented based on the experience with the integration of IIoT into the production process, where a control is provided by the MES system [56]. The compatibility of the solution and integration of IIoT devices is suitably based on the properties of OPC UA standards, which enable multilevel integration, monitoring of time-dependent data, and, also, where the historization of data generated during the production process is ensured [57,58].

That way, it is possible to analyse data in real time, to evaluate, and to adapt control parameters. In vertical integration, the solution would be a device design for the control layer—a technological level, which exceeds the boundaries of a supervision level and an operational level [59]. A flexible control can process new types of signals and improve process control based on these signals. Tools that bring a change in the control view, and are based on Industry 4.0, change the way the control view is oriented [60].

This control view is oriented to perform operations, most of the time realized by a programmable logic controller (PLC), to control based on event-based control. The OPC UA standard presents technical means, methods, and protocols that can be used to integrate IIoT devices regardless of the manufacturer, thus ensuring flexibility and the possibility of improving the controlled parts [61].

The integration of the classic control process with IIoT devices is possible, and with the help of OPC UA, it can bring the benefits of event-controlled processes to existing production lines. The design of an IIoT device is solved based on a proposal where we define what functionality the IIoT should provide, and then a suitable microcontroller is selected based on communication capabilities, performance, and available input and output elements [62].

During the implementation of IIoT devices, it was found that the complexity of the design can lead to an undesirable state where the IIoT device either does not meet the complete requirements or the solution is focused on purely a hardware design, and so its final implementation may not meet the desired result [63,64].

Therefore, before the physical implementation, it is advantageous to produce a model that can provide the function of a digital twin or hybrid software hardware prototype. The digital twin can also be developed and verified by using augmented reality [65], with which it is possible to demonstrate the capabilities of the IIoT device and, as well, to start testing the integration before the final implementation of the IIoT device [66,67].

All these reasons were considered as the basis for the design of the procedure that is presented in this article: a proposal for an IIoT device that provides all of the above requirements.

Historian software, which is part of the AVEVA Wonderware system, can acquire data from the production line. This system includes data storage and compression techniques to provide access to the process, an alarm, and event data. It enables faster decision making, and it is possible to monitor the performance and operations of the production process. Data are collected from one process or from the entire complex of devices in the production line. Data, available from the process control, make it possible to detect the performance requirements and to identify control areas where IIoT devices could be deployed [68,69].

Simultaneously, these data are a source of information for the subsequent integration of an IIoT device directly at the level of the MES system, in the case, when these data are used for production management or they allow one to identify which control units need to be adjusted when deploying IIoT into production. The solution was tested on the FESTO production line.

## 3. Proposal Design

Business process model and notation (BPMN) diagrams were selected to describe the IoT device and identify the processes required to achieve the goal. These were used to determine the initial parameters, inputs, and outputs. The workflow was then constructed. This was converted to a finite state machine, where states and transitions corresponded to the data types and events of the OPC UA standard. This design improved the implementation of the IIoT devices. The advantages are the compatibility with the industry standard, reliability, and guaranteed security of the IIoT device that supports OPC UA communication. These features are not part of standard IoT device designs and are addressed individually. The internal operation and hardware implementation is not limited to the OPC UA standard, but any protocols can be used according to the need by connected components.

The preparation of the design of the functionality of the IIoT device, or its digital twin, could be represented by a basic description, which is based on the BPMN. The first diagram is a general view of the design. In articles [53,54], the authors have shown that it is possible to create a finite state machine model by using BPMN diagrams.

**Figure 6:**
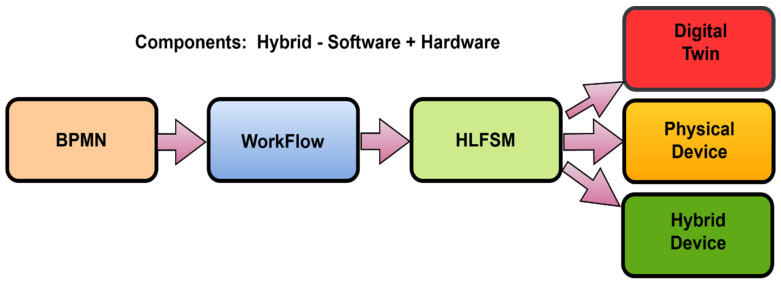
The scheme of the proposal for the IIoT device.

When testing of the noted proposal was performed, an issue was identified where the actual implementation of the high-level state machine (HLFSM), after the transformation of BPMN to HLFSM, did not precisely identify activities and communication that are both needed to occur between the nodes. The scheme of the proposal is shown in Figure 6.

Simultaneously, the creation of one comprehensive BPMN diagram to describe the function of the device is suitable for understanding the principle. However, it may not be suitable for the implementation of the device, because it does not describe all the technical details that are required in the design.

**Figure 7:**
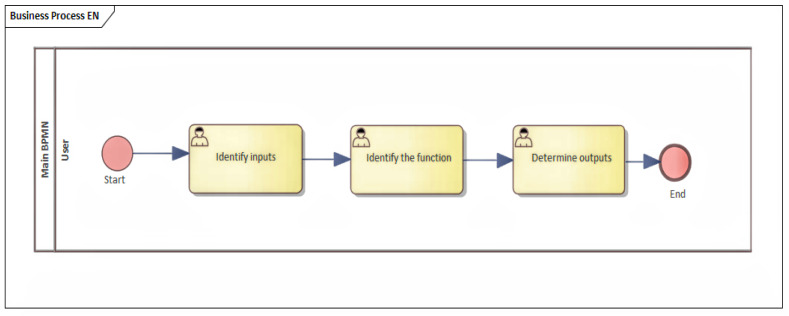
Identification of inputs, outputs, and functions of the device.

Because of this reason, a process was designed where the base is a general description of the process, and the functionality and inputs are identified. Moreover, simultaneously, the outputs that the device can provide are identified, as shown in Figure 7.

**Figure 8:**
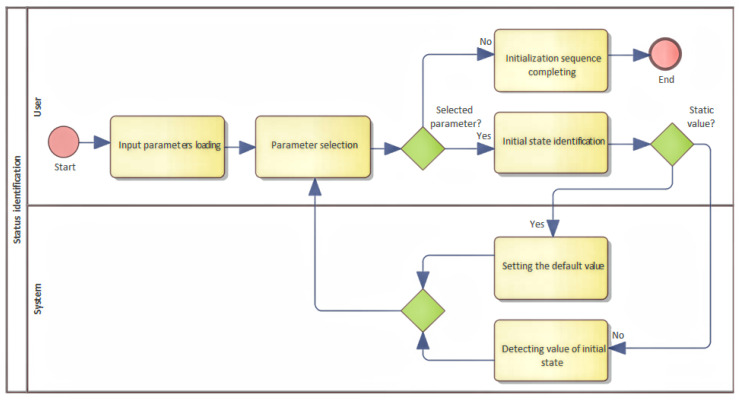
An identification of initial values and states of the device.

The goal of this process is to describe the basic function as well as to understand what a device should implement. In this section, it is advantageous to identify the input parameters that need to be defined, and which initial states need to be detected or set. It is recommended to identify whether the inputs are defined as static, or whether it is necessary to determine some input parameters dynamically during device start. Dynamic parameters can represent internal states that have been created, for example, by previous use, and their values can change use of the device. It could be the height of the liquid level, reading the weight of stored materials, or determining the distance, and so on. In the case of identification of dynamically obtained input parameters, it is necessary to create a separate BMPN diagram for each parameter that describes its functionality. The identification of the inputs and their calibration are both described in Figure 8.

The identification of inputs and their classification at the device start is equivalent to the startup sequence that is performed each time the device is turned on. The aim of this simple description of inputs and outputs was to break a more complex process down into subprocesses, because that way they are better understood.

**Figure 9:**
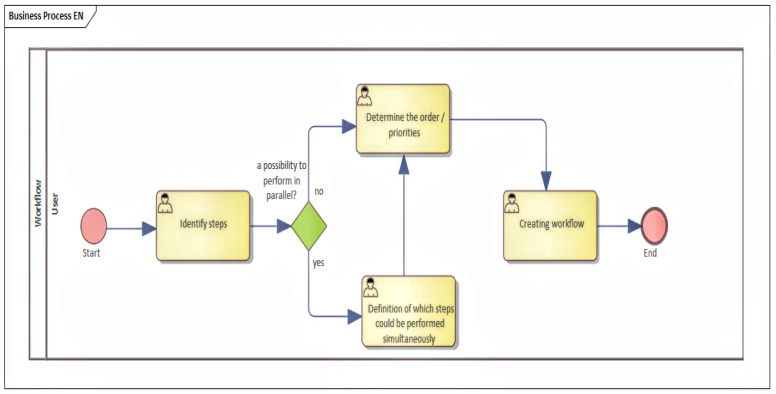
Identification of activities and creation of steps.

It was necessary to describe the functionality right after the device was initialized. The result of the analysis was an overview of each step that led to the achievement of the required outputs (or design results). The design of the process is shown in Figure 9. The analysis of functionality was, of course, based on the nature of the device requirements. It is advantageous to include the existing data in this analysis, where the suggested devices are to be integrated. For IIoT needs, there were data available from the production line, which were obtained from the supervisory control and data acquisition (SCADA) or MES systems. These data were helpful in identifying the steps that needed to be taken. It is advisable to understand the existing process again or to define the goal that needs to be achieved. An identification and an atomization of steps were both considered as the base, which, after evaluating and determining their feasibility, enables creation of a tree of dependencies of individual steps. In this form, it is possible to visualize the individual steps in a tree that represents the workflow. Here, it is clearly possible to identify how the goal is achieved, and, simultaneously, it represents a visual process of the internal functioning of the device.

**Figure 10:**
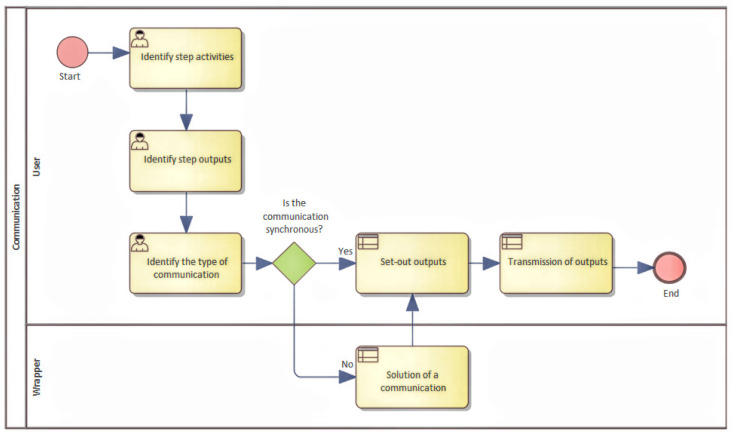
An identification of activities and outputs.

If necessary, and if some steps would present a more complex problem, it is possible to create a nesting and thoroughly break the step down. This step could be replaced by another tree of steps that defines the workflow. From the existing data of the production line, it is possible to determine whether there is a reason to include time-critical execution of operations in the analysis or to identify values that can act as data for device inputs or outputs, which the device provides to improve the production process; by delivering data from its inputs, it affects the existing control members of the production process, as shown in Figure 10.

The analysis of communication and data exchange between steps is another part that potentially represents an area that needs to be specified. Here, it is necessary to classify the type of communication. There are two possible types of communication: synchronous or asynchronous. The simplest case is synchronous communication, where data are exchanged between the steps. In the case of asynchronous communication, it is necessary to define how communication will be implemented.

**Figure 11:**
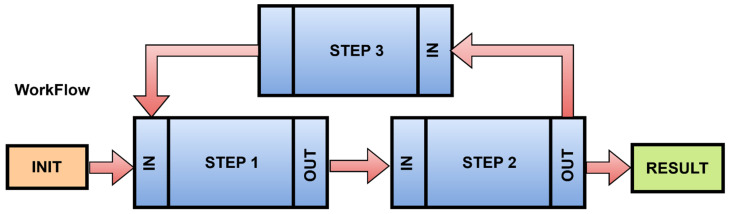
A scheme of drawn steps and their dependencies—workflow.

A workflow that can be assembled from the steps to be performed consists of basic parts as shown in Figure 11. The communication between steps is indicated by arrows.

An init process was introduced to the STEP1 input. The init process performs the operation or action that is defined, and the result is sent through the output function of the step to the input of the next step. This workflow modeling system graphically displays the data flow, and it allows for the identification of the method of communication. Each step represents an action. Communication between steps is handled by protocols. It does not have to be only the OPC UA protocol. It may internally use protocols that provide the appropriate type of communication. For IIoT devices, standards are recommended, which provide internal communication at the level of inputs and outputs. These standards are shown in Table 1. The choice of protocol is influenced by the specific need for communication.

Depending on the type of interface that needs to be implemented, it is possible to select an appropriate protocol type. Usually, the sensor or component that we should connect is already specifically equipped with a port, and it requires a specific protocol. It is possible to show how the communication at the IO level will occur for the workflow. If it is possible to choose from application-level protocols, it is possible, without any problems, to combine these types of protocols, even if the primary purpose of these application protocols is to ensure communication toward the environment.

Each IIoT device communicates with the environment, and it depends on the level of the IIoT device which tasks will be performed within the local implementation and which tasks can or must be submitted for processing to external sources. In cases where data are submitted to a cloud environment that has greater computing power, or the cloud environment is oriented to a specific type of task, such as machine vision, machine learning algorithms for searching for patterns in data or processing, and evaluating complex data by using big data techniques. An overview of application-level protocols is shown in Table 2.

Specific needs of IIoT devices are also represented by application protocols. Specific needs of industry protocols may also represent increased demands on the hardware and software parts of the IIoT device. For realtime device deployment, there would be a question whether an IIoT device can perform this function.

Depending on the use and implementation, it is possible to divide devices into levels according to their ability to perform the function independently or to rely on external processes that can be performed on external devices or in the cloud.

A basic design of IIoT is implemented as a complex unit that can perform the function independently. Data, generated from this device, are stored and prepared for processing by the IIoT device itself. The analytical part is also implemented and performed directly in the IIoT device. If the design identifies the need to store data, and the capacity of the proposed device is not sufficient, it is advisable to store data externally or in the cloud. The same applies to the processing of results, the implementation of analytical methods, or machine learning, where it is possible to use external resources, for example, cloud computing. This part may be a critical part of the industry from the perspective of data security or sensitivity. From a technical point of view, however, there is nothing to prevent them from being implemented in this way.

**Figure 12:**
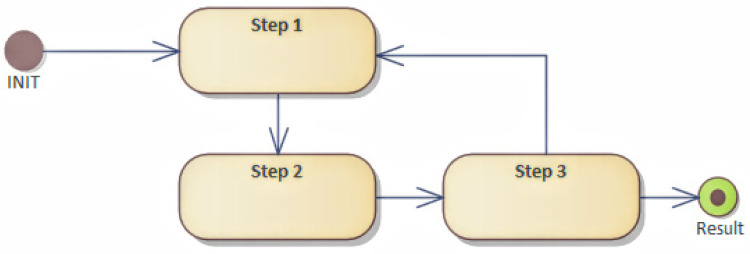
High level finite state machine.

An abstraction, with the help of workflow, helps streamline data flow as well as it helps with performing steps. However, this representation does not correspond to the programming approach. Therefore, it is appropriate to transform the workflow into a HLFSM, as shown in Figure 12.

This transformation consists of defining states and their context. The state can be represented by a class, and the action can be represented by a method. Each state that the device acquires has a defined event that affects which state the system is in. An event can be considered as an internal or an external event. The state change is called a transition in a system. Typically, the state transition in the system corresponds to one transition and to one event. An event is an internal or external change, such as a button press, a click, or an expiration date, which can change the state of an object, leading to a transition state in the system.

This system typically corresponds to only one pass of a given event. It is possible that there is more than just one transition. Thus, several conditions can change immediately. The transition represents a change from one state to another, which represents the state machine‘s response to the occurrence of a particular event. The transition can be controlled or conditional. In such a case, it is determined whether it is possible to transition to another state, and whether the event should continue, or if it will be rejected and subsequently stopped. The state transition may only be associated with one or more actions to be performed when the state changes. An action defines an activity that is performed, and it can be associated with more than one transition, and a transition can have more than one action. The action can also trigger an event that triggers another transition.

The transformation from the workflow is relatively simple. A step in the workflow represents an activity that is performed. The workflow describes steps that change the state. If the state does not exist, it must be created. The sequence of steps represents the gradual interconnection of transitions between states. By traversing the entire workflow, it is possible to complete all transitions and define states, and that way record events, which represent a change in states and transitions in the system. In the case of parallel execution of steps, these are actions, which can initiate multiple transitions or events immediately. In this way, it is possible to identify the internal behavior of the model, and, simultaneously, the workflow; it can be beneficial in terms of data exchange and protocols used.

In the first way, by creating a state machine, it is possible to proceed according to the OPC UA information model.

**Figure 13:**
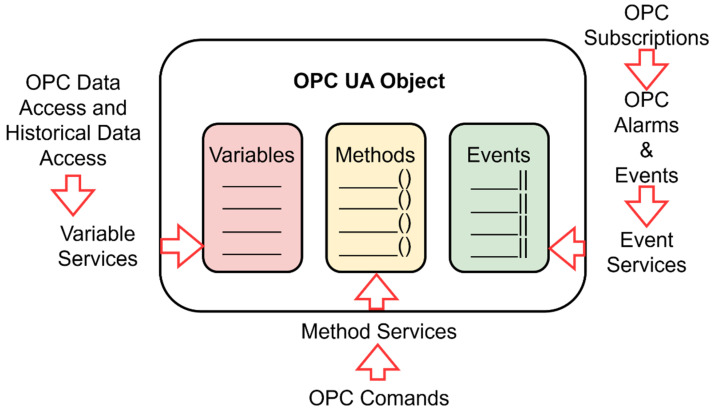
OPC UA object.

The state machine is the basis for the OPC UA object, as shown in Figure 13. The mapping of the finite state machine to the OPC UA object is as follows. Variables are mapped to the object in the variables section. Variables can use existing types according to the OPC UA standard or they can be defined by the user. Each state and its transition is represented by a method. One state can comprise multiple methods. If one state has multiple methods, it may or may not be possible to change the state automation so that each function is represented by a new state, depending on the system requirements. Transitions between states represent events. An event can be used to control or monitor a production process.

By transforming the state automaton into an OPC UA object, all available methods and events and the current values of the set variables are provided. This meets the integration requirements for the industry. Simultaneously, the state automaton can be transformed into a comprehensive information model representing a description of the device and its internal workings in the OPC UA, which a standard design procedure for IoT device development would have to create separately.

The selection of hardware parts and subsequent testing can occur in several ways. One possibility is to implement an IIoT device completely as software and replace the sensors or the necessary hardware parts with values, which are assumed or obtained by specific historical data from the production process. The first result can be a software-implemented digital twin, which represents the functionality of the IIoT device. There it is possible to test the integration and to monitor the impact of inclusion on the production process and perform simulations to identify or to evaluate the significance of the intended change.

The second way is to create an IIoT device as a hybrid device, where the hardware parts will be partially connected. However, it may not be the final solution. For example, sensors may be connected that perform the expected function, but their connection may not correspond to the protocols and ports that will be used in the final connection. In this way, it is possible to replace some parts, but also to create a functional prototype, which can be used as a test solution that can reveal potential problematic parts of the design. However, it is possible to implement communication layers at the application level and have an interface ready that can be reused in the final product of the IIoT device. The advantage is the ability to test the external parts of the application logic, which can be implemented in the external sources or executed them in the cloud.

## 4. IIoT Device Proof of Concept

**Figure 14:**
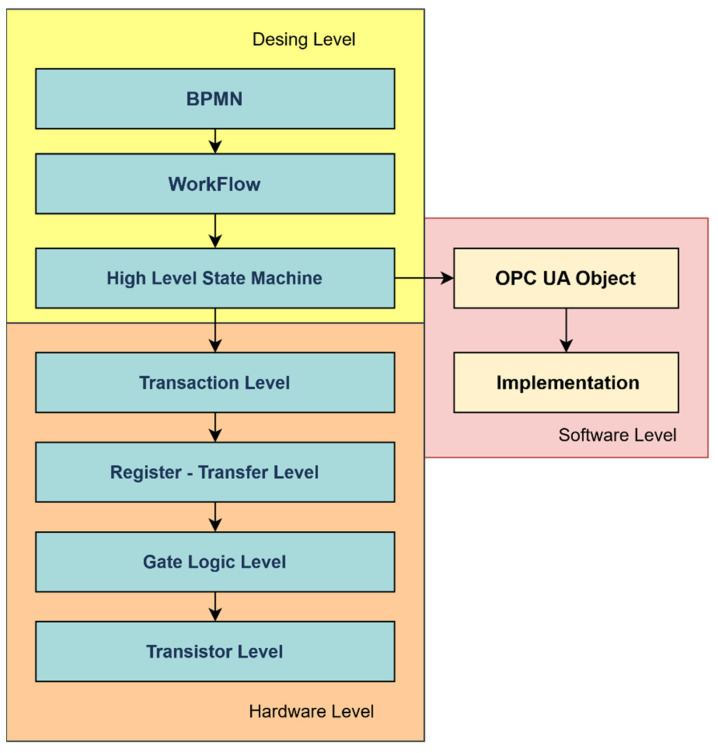
A procedure of an IIoT device design.

A procedure of an IIoT device design is shown in Figure 14. It is based on the business process, which can be used to analyse and to identify requirements by using the proposed procedures. The inputs and outputs that the device should contain are then also detected.

**Figure 15:**
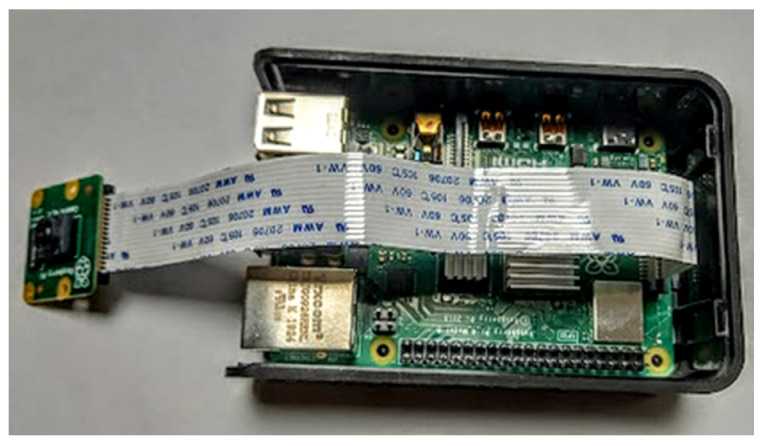
Proof of concept IIoT device.

The proposed IIoT device was implemented using a Raspberry Pi 4B shown in Figure 15. The implementation was completed in Python programming language.

**Figure 16:**
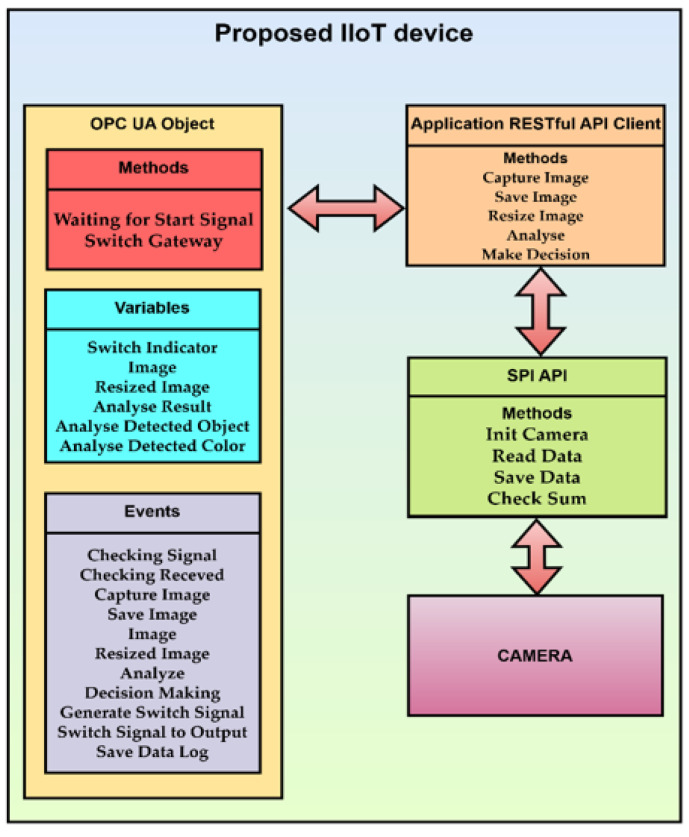
The block diagram of the proposed IIoT device.

The block diagram of the proposed IIoT device is shown in Figure 16. The block diagram shows the structure of the proposed IIoT device. It consists of methods and events of OPC UA object, methods of application RESTful API client, methods of SPI API, and a camera.

The production line was a functional model where the filling of liquid or bulk material occurred. A robot was located in the recycling section, where bottles that were poorly filled, incorrectly closed, or filled with incorrect contents were being cleaned. In this part of the production line, the incorrect placement of bottles in the correct container was detected, and, as well, the design of IIoT device will be demonstrated.

**Figure 17:**
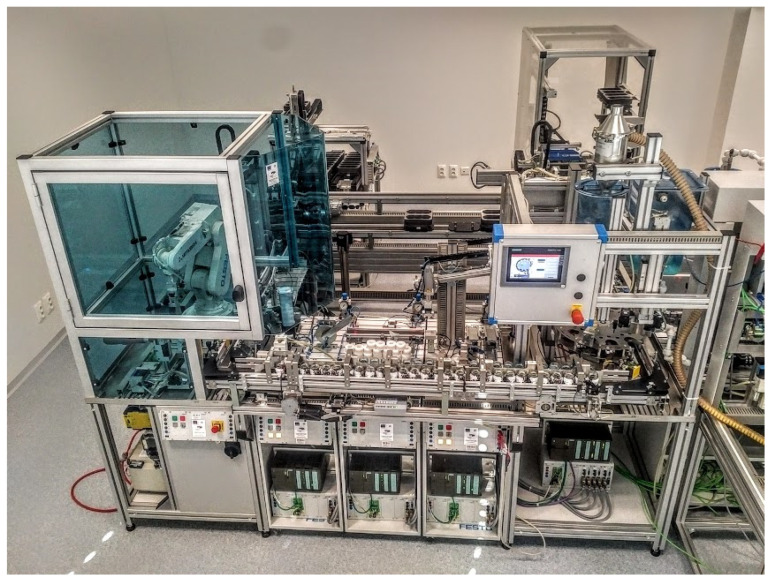
The model of production line used for proposed IIoT device testing.

The testing of the proposed IIoT device was performed on a production line shown in Figure 17.

**Figure 18:**
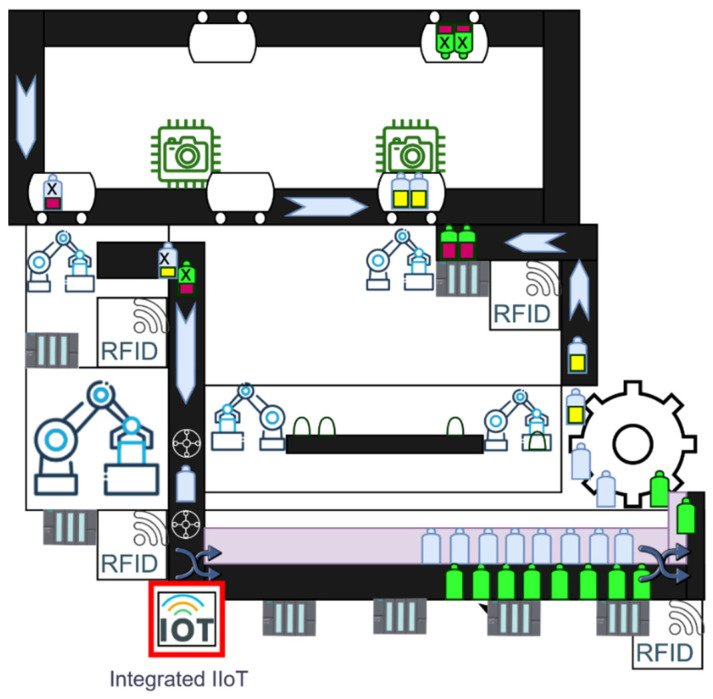
An integrated IIoT device in the production process with a bottle detection function.

The task of a suggested IIoT device was to detect the bottle at the outlet of the recycling station. There were two types of bottles. The first type of bottle was blue and was used for liquids, and the second bottle, which was intended for bulk materials, was green. The suggested IIoT device was to identify the passing bottle and to accordingly send a signal to the gate, which correctly put the bottle back into the filling process. The schematic location of the suggested IIoT device is shown in Figure 18.

**Figure 19:**
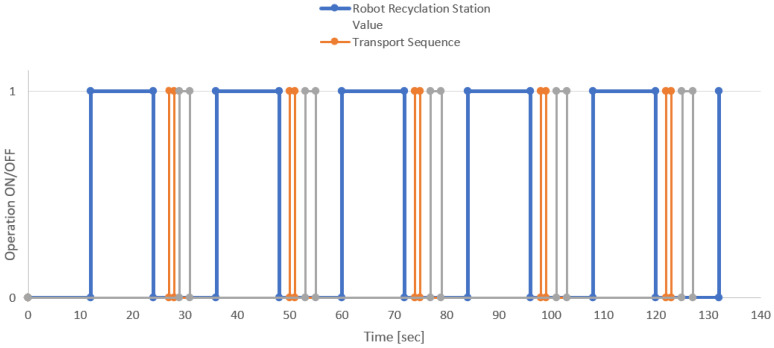
Length of operations of the production line components.

Data from the production process, which are shown in Figure 19, were used to analyse the process. The blue line shown represents the robot’s operating times in a steady production process. The bottle recycling represents the total time required to aspirate the material. The robot operation started in the 12th second, and it was completed in 24 s. This time can be variable because it may not correspond to a fixed period, even though in this graph the operations lasted almost as long.

The essential information is the edge of the blue line, which represents the end of the robot recycling. This triggered the movement of the conveyor belt, and the orange mark indicates the passage of the bottle to the place where it leaves the recycling sector. Such impulse and its terminating edge represent a place where bottle type detection can be performed. In this type, this state represented an event that triggers an IIoT device. The conveyor belt was stopped, and it was possible to scan the bottle, and the bottle identification was started. The gray line represents the operation and duration of the IIoT device operation.

Table 3 lists the inputs and outputs that the IIoT device should provide.

The starter sequence was determined below. In the test device, the first init process was started, which was also labeled as reset. The reset was used for the default setting of values. In the case of this device, it may be a matter of resetting the number of frames and setting the system time or configuring and connecting external sources as well. The init function of the IIoT device is described in Table 4.

**Figure 20:**
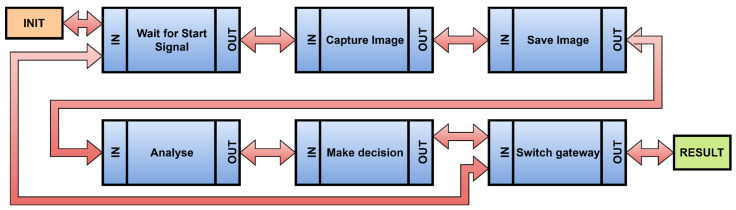
A workflow of the IIoT device.

An identification of the steps for how the device would perform its operation would be the following. Schematically, the illustration is shown in Figure 20. The first step is the start, and it is identical to the init process. After turning the device and its start on, the device is ready in a state, where it is awaiting a command. The first step is to check that the action is running. The second step is to capture the image information. This step has an input, which represents the signal supplied from the previous step and, simultaneously, an input that represents a sensor, in this case a camera. The next step may be to save the image. The following step is to prepare the scanned image for an analysis. The image is then analysed, and the type of bottle is evaluated, when the switching of the gate on the production line is ensured as well.

**Figure 21:**
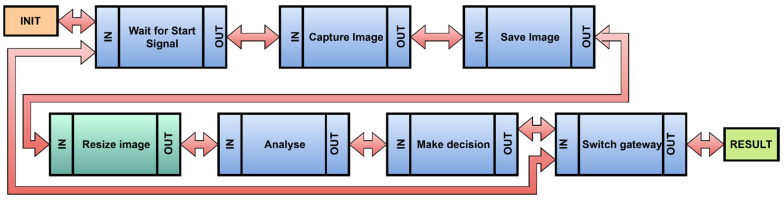
Missing steps added into workflow.

Each step can be described more thoroughly. An image analysis can be represented by another workflow. One step in the workflow is responsible for one action, which may not be the case for image analysis, and so multiple actions must be performed. Therefore, it is necessary to divide this step into two steps, for example, to reduce the image, and then perform an analysis. The change of the added step is shown in Figure 21, where the added step is distinguished by a color.

After creating a workflow representation, it was possible to proceed to the creation of a state machine. By going through the steps, it was possible to create the following state machine, shown in Figure 21, where state transitions can be seen.

The input and output were now shown at each step. It was possible to prepare a table of protocols that would be used for communication, if the connection between the steps required it. Table 5 shows several steps that were performed in this way.

Every step defined its own protocol for communication. In this test scenario, OPC UA was chosen for integration and handled the external communication with the OPC UA client. The SPI protocol was used to handle communication with the camera. The camera was part of the device. This was required by using a camera device, and it can be different. The next steps were implemented in cloud or external API, and there was an HTTP call for RESTful API, which can process a specific action or execute methods, which can provide functionality by need. Each step can be performed in the IoT device and processed internally if needed, and there are enough resources.

The configuration of the protocols can be completely different from the focus and the chosen implementation goal. Here, a OPC UA protocol was selected, where the IIoT device would be the OPC UA server, which provided all the necessary methods.

For the implemented prototype, image processing in the cloud system was used, and an API interface was implemented, which evaluated the captured images. In the case that the IIoT device will be independent and will process all activities, all steps and protocols of the proposed device must be adapted to the given hardware specification. For example, image storage would be performed on a local disk or by using an API call that would implement a physical IO port, bus, serial memory, and so on. In the workflow part, the input and output are written at each step.

Again, it is possible to describe which step will send the information and in what form, if necessary. However, in most cases, this part is linked to the selected type of communication. In the case of this article, the OPC UA was a server, and it was not just one type of message.

In the next part of the table was an HTTP application protocol, which was the protocol for the API calls. The RESTful API was chosen, which is easy to implement and can contain data structures in the form of JSON and work with attachments, which was necessary in this case, as the image information was sent outside the IIoT device.

It is possible to define what type of call must be made to send data. For the save image step, the data were sent by a PUT method, and it was possible to attach metadata of the image and attach the image itself. For a resize image, it may be a POST call. In the next step, analyse, it may again involve a POST call.

By calling make decision, it determined, according to the configuration of the analysis, how the gateway would be set. The analysis can send several parameters to its output. Here, for simplicity, two were handed over.

The first parameter was the identification of the object, to what percentage this object was identical with the learned tensor flow model, and the second parameter was the evaluation of the bottle type, which was determined based on the color combination.

The limit values of the decision-making system were determined for evaluation and correct decision making. In the state machine, as will be shown, another step was finally added, which recorded the data that were processed by the IIoT device.

These parameters were also transferred by using the OPC UA protocol to the MES system, where they were processed by the Historian process and stored for possible further evaluation of the production process.

**Figure 22:**
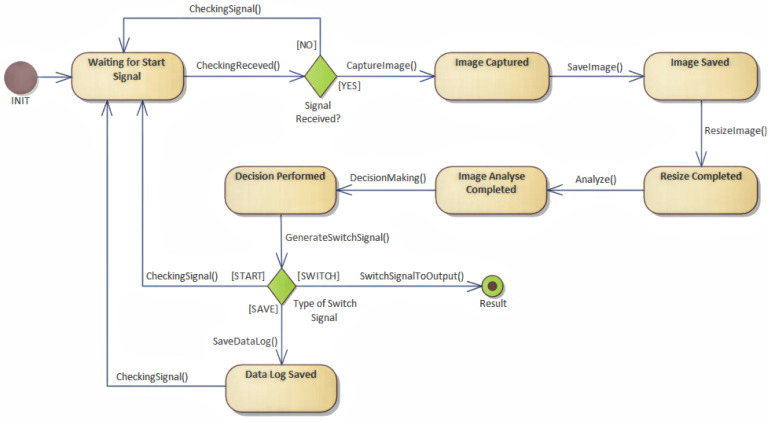
Transformation to high level finite state machine.

Workflow processing on HLFSM was done by going through the workflow steps, and so it was possible to generate and to specify a state machine and define states and transitions between them, as shown in Figure 22. For recording or log level, it would be possible to record the state transitions for each state, even with the data that are affected, and thus obtain the complete internal functioning of the device. Simultaneously, according to OPC UA, it is possible to provide an internal functionality of an IIoT device in the form of a state machine. The result of the notation is a format representing the application logic, and so it is possible to access both software and hardware implementations.

## 5. Discussion

A design of the solution, which was already prepared as HLFSM, can be implemented with various tools with which it is possible to implement a digital twin. The design allows monitoring of internal states and changing parameters that can be tested before physical implementation. It is recommended to follow the standard application protocols for device input and output. Here, the OPC UA standard can be used for real implementation.

**Figure 23:**
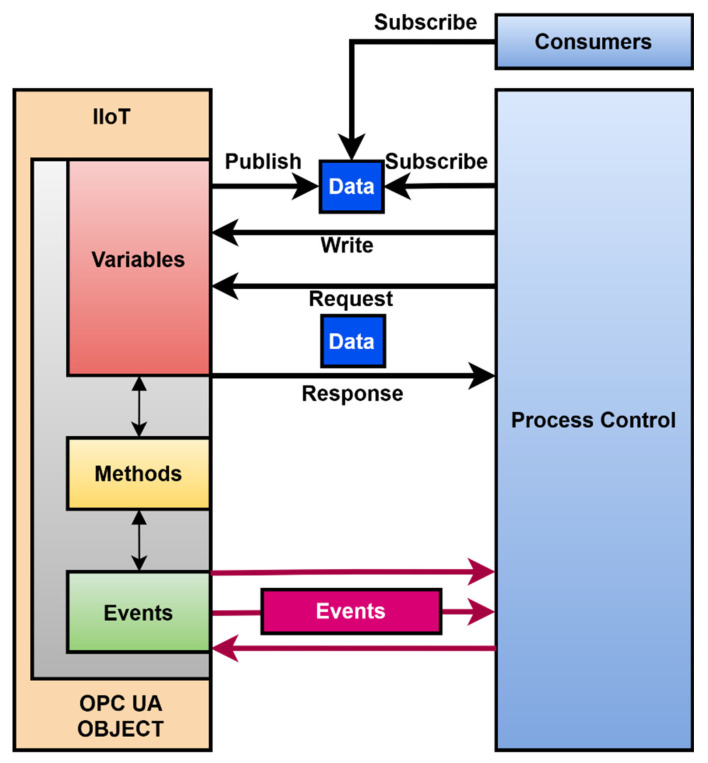
IoT device design and integration options.

OPC UA object provides all technical advantages for a production line. There are more standardized ways of connectivity, which can keep the original concept of IoT devices. There is compatibility with MQTT with user datagram protocol (UDP) transport. It supports binary transfer as well as JSON structers and OPC UA publish subscriber connectivity. There is an option to use transport layer security (TLS) for data as well to use the public subscribe security model of OPC UA, as is shown in Figure 23.

Data can be acquired by more consumers. Process control can receive events and use them for better process control. Data blocks in the OPC UA object can be specified for bidirectional communication. This can be used to configure the device. However, this feature provides a much more efficient distribution of processing tasks to more powerful external resources. The design can be partitioned in this way so that the same functionality is performed in the IIoT or another using external computing power.

This implemented functionality could remain the same for the digital twin and for the final design of the IIoT device. Digital twin technology, along with the company’s analytical tool, will provide the opportunity to analyse the impact of IIoT devices on the production process. If the MES system allows connectivity, then the digital twin can also be involved in the production process. This also applies to simulation software. The protocol can be chosen according to the technologies being used. OPC UA-CloudLibrary is ready to provide a function that can create a digital twin based on cloud technologies.

The created state machine representation is much closer to integration in industry with PLC control elements. The proposal can also be applied to the development of applications for PLC devices. The test was performed using an implementation in the Python language, where a state machine was implemented by using a library with OPC UA ability. This implementation also served to create a hybrid solution with connected hardware elements, which were replaced in the digital twin and connected via specific physical ports and protocols.

The final hardware implementation of the device will follow the additional layers that followed the creation of the state machine, as shown in Figure 22. Each state of the automaton can be further processed, according to standard software development, where it is possible to produce a process model, a domain model, an information model, a service model, and a functional model. An additional one is the UML state machine model, which is the basic proposal for an OPC UA object.

The suggested proposal allows the implementation of a detailed hardware level. It is possible to identify and select suitable components and to design a printed circuit board [PCB]. In the case of complete hardware design and implementation, it is possible to proceed to the implementation of register transfer level (RTL) at the lowest level, and thus design essential logic circuits. Here, it is necessary to follow the design process and to apply it repeatedly until the state machine level is reached, which is suitable for the design of communication at the level of hardware registers, and logical operations that can process control signals. Further research into IIoT devices will head in this direction.

### Limitation of the Work

The proposed method has the disadvantage of not being able to verify the hardware compatibility of the components by referring only to the use of protocols in the workflow. This limitation has been partially highlighted in the identification of the workflow steps and their communication with each other. A solution to this limitation may be to group the workflow steps, so that they are represented in the state automaton using a single state. Here, loss of information and events or executed functions is possible. This part can be challenging when debugging an IIoT device. Regardless, in the final hardware implementation this may not be a sufficient solution.

Another issue can be too much fragmentation of the workflow steps, which leads to overloading the device represented by the OPC UA object. It is recommended to include in the variables the performance tracking of the resources available to the IIoT device itself, e.g., the central processing unit (CPU) load, memory usage, temperature of components, etc. The solution to this IIoT device issue is possible by delegating tasks to external resources or the cloud. This may represent a significant intervention in the design of the IIoT device. In terms of a digital twin, there is no definition of communication protocol for simulation software. This can be solved by choosing the correct protocol or data structure that is supported by simulation. This can be solved by an OPC UA standard in the near future. Because the design is intertwined with an OPC UA object, there may be a disadvantage of using other protocols that are excluded.

## 6. Conclusions

The prepared proposal was implemented in laboratory conditions. The classic development of the IIoT device, and the preparation of the digital twin, proved to be nontransparent during the implementation in the industry. The representation of PLC control elements and the implementation of control, by using the MES system, showed shortcomings that have been described in this article.

Therefore, a procedure was prepared using business processes and BPMN diagrams to identify goals and then to build a workflow to be performed by the IIoT device. In this section, there was an advantage of identifying protocols and their levels, assigning steps and identifying their computational complexity, and determining which steps will be implemented locally in IIoT devices and which steps will be implemented by using external calls on more powerful devices or in the cloud. Simultaneously, the workflow, which was created during the analysis, described all the steps that need to be performed, based on which it was also possible to identify dependencies of individual steps.

By transforming the sequence of steps into a state machine, the solution moved closer to the standards of Industry 4.0. The OPC UA object created was represented by methods, data, and events. All of these could be designed based on the state machine model. Mapping and all definitions of states, actions, and events were easily achieved. The advantage of this solution was to obtain much more information about the internal functionality of the IIoT device.

The noted proposal is compatible with devices that can use the OPC UA standard as a communication protocol to ensure wide compatibility of the solution. The use of data from the existing production process also defines the communication and time limits that must be achieved in the implementation of IIoT devices.

A verification of the functionality and impact of the intended implementation was possible with use of HLFSM. The HLFSM is easy to implement in software and hardware. Simultaneously, the state machine can be used to generate data that can be analysed together with the existing data of the production process.

The hybrid solution used a software implementation of a state machine and integrated physical elements. Based on this, the final realization of the IIoT device was possible. All attributes were aimed at ensuring that the IIoT device was clearly designed, and it could demonstrate the impact of its integration on the production process.

## Figures and Tables

**Table 1:** Overview of IO protocols for IIoT devices.

Protocol	Duplex	Communication Type
UART	full-duplex	Asynchronous
SPI	full-duplex	Synchronous
1Wire	half-duplex	Synchronous
I2C	half-duplex	Synchronous
CAN	CAN	BUS Asynchronous/Synchronous base on time
SDIO	SDIO	BUS Asynchronous/Synchronous base on time

**Table 2:** Overview of application protocols for IIoT devices.

Level	Protocol Type	Communication Type
HTTP	Request/response	Asynchronous
MQTT	Publish/subscribe	Synchronous
OPC UA	Publish/subscribe	Synchronous
AMQP	Publish/subscribe	Synchronous
CoAP	Publish/subscribe	Asynchronous
DDS	Publish/subscribe	Asynchronous
Web Sockets	Bi-directional	Asynchronous
S7comm	Bi-directional	Asynchronous
MOD BUS	Bus bi-directional	Asynchronous
PROFINET	Isochronous real time	Isochronous
PROFIBUS	Bus Isochronous real time	Isochronous

**Table 3:** Inputs, outputs, and a description of a basic function of the IIoT device.

Type	Name	Description
Input1	Starting	Inputs, outputs, and a description of a basic function of the IIoT device
Input2	Camera	The input is used to capture an image
Output1	Gateway	The output generates and secures a gateway switch
The basic function	IIoT	Detection of a bottle type

**Table 4:** Init function of IIoT device.

Type	Name	Description
Init 1	Reset	Settings of initial values and parameters
Init 2	Performs the entire program run for the first time	Detection of initial states

**Table 5:** Description of protocols for individual steps.

Step	Protocol	Description
Waiting for Start Signal	OPC UA	The MES system delivers a message to run the action on the IIoT device.
Capture Image	SPI	SPI Camera. This results from a hardware connection.
Save Image	HTTP	Outside storage of the IIoT device, save—REST call PUT method.
Resize Image	HTTP	Data are stored on external storage, resize—REST call POST method.
Analyse	HTTP	Data on the external storage, analyse—REST call POST method.
Make a decision	HTTP	Data are stored on external storage, decision—REST call POST method.
Switch gateway	OPC UA	Message to the MES system in order to set the gateway to the desired state.

## Data Availability

Not applicable.

## Abbreviations

**Terminology**	**Description**
IoT	Internet of Things
IIoT	Industrial Internet of Things
OPC UA	Open Platform Communications United Architecture
UA	United Architecture
FSM	Finite State Machine
HLFSM	High-Level State Machine
API	Application programming interface
HW	Hardware
SCADA	Supervisory control and data acquisition
MES	Manufacturing execution system
IN	Input
OUT	Output
MQTT	Message Queue Telemetry Transport
BPMN	Business Process Model and Notation
UART	Universal asynchronous receiver-transmitter
SPI	Serial Peripheral Interface
1Wire	One Wire
I2C	Inter-Integrated Circuit
CAN	Controller Area Network
SDIO	Secure Digital Input Output
HTTP	Hypertext transfer protocol
AMQP	Advanced Message Queuing Protocol
CoAP	Constrained Application Protocol
DDS	The Data Distribution Service
S7comm	S7 Communication
MODBUS	The data communications protocol
PROFINET	Industry technical standard for data communication over Industrial Ethernet
PROFIBUS	Fieldbus communication in automation technology
CPU	Central processing unit
PLC	Programmable Logic Controller
